# Living with Parkinson’s disease: disease and medication experiences of patients and caregivers

**DOI:** 10.1080/17482631.2021.2018769

**Published:** 2022-01-02

**Authors:** Yi-Wen Chen, Chu-Yun Huang, Jo-Hsin Chen, Chi-Lien Hsiao, Chien-Tai Hong, Chen-Yu Wu, Elizabeth H. Chang

**Affiliations:** a Department of Clinical Pharmacy, School of Pharmacy, College of Pharmacy, Taipei Medical University, Taipei, Taiwan; b Department of Pharmacy, Shuang Ho Hospital, Taipei Medical University, New Taipei City, Taiwan; c Department of Pharmacy, Wan Fang Hospital, Taipei Medical University, Taipei, Taiwan; d Department of Neurology, Shuang Ho Hospital, Taipei Medical University, New Taipei City, Taiwan; e Department of Neurology, School of Medicine, College of Medicine, Taipei Medical University, Taipei, Taiwan; f Research Center for Pharmacoeconomics, College of Pharmacy, Taipei Medical University, Taipei, Taiwan

**Keywords:** Parkinson’s disease, medication therapy, patient, caregiver, qualitative research, medication experience

## Abstract

**Purpose:**

Symptoms and medication use in patients with Parkinson’s disease (PD) affect the quality of life of patients and caregivers, yet prior research seldom focused on their experiences with medications. This study explored comprehensive living and medication experience from patients with PD and their caregivers.

**Methods:**

Patients diagnosed with PD for ≥2 years, with or without their caregivers, were recruited from an outpatient clinic in Taiwan. Semi-structured in-depth interviews were conducted based on the Common Sense Model. A qualitative content analysis was used to identify salient themes from verbatim transcripts.

**Results:**

In total, 15 patients and eight caregivers were interviewed. Five themes were derived: (1) symptoms and help-seeking behaviours before a diagnosis, (2) emotional impacts and life adaptations after a PD diagnosis, (3) life affected by medications, (4) experiences of caregivers in taking care of PD patients, and (5) communication between doctors and patients.

**Conclusions:**

Patients frequently adjusted their daily schedules to live with PD and the medication side effects. Caregivers struggle to overcome caring burdens and to stay positive to support patients. More attention on providing medication information, mental support, and communication between stakeholders is needed to improve the quality of life of patients and caregivers.

## 1. Introduction

Parkinson’s disease (PD) is a prevalent neurodegenerative disease especially in elderly populations (Tysnes and Storstein, 2017). Common symptoms of PD are characterized by tremors, rigidity, bradykinesia and postural instability; in addition, non-motor symptoms such as sleep disturbances, autonomic dysfunction, psychological distress and cognitive impairment have also been reported in PD patients. Although treatment is unlikely to cure patients of PD, anti-parkinsonian medications are prescribed for patients to control their motor symptoms and reduce their physical disabilities. However, medication side effects, such as motor fluctuations causing PD symptoms to return before the next dose or other movement complications, often occur with long-term medication use (Shin and Habermann, 2016a, 2016b).

Medication adherence is significant for symptom control and treatment outcomes. Adherence rates of anti-parkinsonian medications ranged 33%~98% (Shin and Habermann, 2016b). Non-adherence to anti-parkinsonian medications is associated with poor control of PD symptoms, which might lead to increased risk of movement complications, social burdens, and healthcare costs, as well as a reduced quality of life (QoL) (Straka et al., 2019, 2018). Several aspects of factors influence non-adherence in this population were found, such as cognition disorders, financial issues, lack of a caregiver, inadequate knowledge, and a low QoL (Shin and Habermann, 2016b). However, little is known about patients’ subjective medication experiences and medication beliefs after taking anti-parkinsonian medications.

Both PD patients and their caregivers are faced with multiple difficult aspects. On the one hand, patients are faced with impacts on physical functions and psychosocial adjustments along with PD progression, generating the need for support and resources from healthcare institutions and society (Liao et al., 2013; Maffoni et al., 2019). Mobility dysfunctions, social embarrassment, and uncertainty about the future all influence patients’ social roles, health awareness, and QoL (Fox et al., 2017; Theed et al., 2017; Thordardottir et al., 2014). A qualitative study demonstrated patients’ resources and hindrances of living with PD over time. Patients mentioned concern about bodily changes, loss of autonomy, and roles of the family and social networks after a diagnosis (Maffoni et al., 2019). On the other hand, caregivers of PD patients play an influential role in patients’ lifestyle adaptations. Yet caregivers face heavy burdens due to their care responsibilities. Their unmet needs and conflicts in caring could lead to anxiety, depression, and a reduced QoL (Fox et al., 2017; Perrin et al., 2019; Tan et al., 2012; Theed et al., 2017). Particularly when patients’ cognitive abilities deteriorate, care burdens and result in caregivers’ frustration, a sense of alienation from patients, and isolation from society (Corallo et al., 2017; Fox et al., 2017; Tan et al., 2012; Vatter et al., 2018).

With regard to medication experiences, few studies mentioned issues about medication-taking, such as anxiety and stigma of side effects, as well as caregivers’ burdens regarding medication scheduling and administration (Corallo et al., 2017; Liao et al., 2013; Tan et al., 2012). A qualitative study investigated experiences and decisions of PD patients when initiating anti-parkinsonian medication therapy (Shin & Habermann, 2016a). The acceptance of taking medications was based on trust in healthcare professionals and a patient’s desire to continue working. Some patients tended to delay the initiation of medications due to concerns about the early onset of side effects and a shortened response to medications as PD progresses. Nevertheless, subjective experiences after patients have been taking medications for longer periods remain to be characterized. In addition, the meaning of medication use in this population is not well understood. Therefore, this study aimed to explore comprehensive experiences of PD and medications use from both patients’ and caregivers’ perspectives.

**Figure 1.**
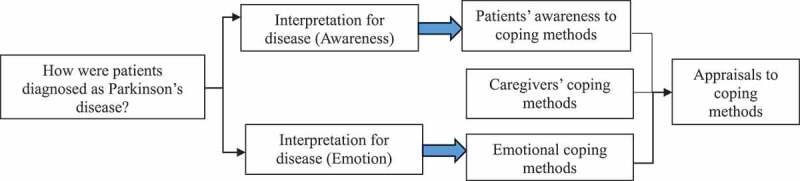
Research framework.

The framework of the present study was informed by Leventhal’s Common-Sense Model (CSM) (Leventhal et al., 2011) to examine PD patients’ subjective awareness, emotional actions, and both patients’ and caregivers’ experiences of coping with the disease (Figure 1). It defines patients’ self-regulation in the process of illness representation, coping strategies, and appraisals. The causes of a disease, its controllability, and symptoms shape patients’ illness representation, which determines their coping strategies—including attitudes towards the illness, emotional expressions, and care-seeking behaviours. After implementing coping strategies, patients appraise the strategies by improvements in symptoms or treatment outcomes. Moreover, since the coping strategy is usually related to their caregiver, we also investigated coping methods of caregivers.

## 2. Methods

### 2.1. Design

This was a qualitative study using semi-structured, individual, face-to-face interviews to explore the living and medication experiences of PD patients and caregivers that was conducted from September to December 2016.

### 2.2. Participants

Patients with PD who had been diagnosed for at least 2 years, were aged 20 ~ 85 years, who were current anti-parkinsonian medication users, and were able to communicate in Mandarin, were recruited from a single outpatient clinic in Taiwan. Caregivers including family members and care workers who were familiar with the patients’ living patterns, were aged 20 ~ 85 years, and were able to communicate in Mandarin were invited to join the same interview with their patient.

### 2.3. Procedures

Eligible patients were identified at neurological outpatient clinics by physicians, and patients were provided with a study information sheet. Patients who were willing to join the study were referred to the interviewer. Patients were interviewed in a medication counselling room at the hospital, with or without their caregivers being present. After acquiring informed consent, participants were asked to fill out a demographic questionnaire before the interview. The interviewer was a pharmacy student with knowledge of PD medications, and special training in qualitative methods and in-depth interview skills; a pharmacist in the consultation room was responsible for providing professional advice after the interview. Based on a semi-structured interview guide (Appendix), pilot-tested and revised further before the study, the interviewer motivated interviewees to provide vivid descriptions by reflecting on their personal experiences in a relaxed atmosphere. The interviewer deeply probed to better understand patients’ feelings and specific experiences. Participants were given NT$ 100 (roughly US$ 3) as an incentive for their participation. All interviews were recorded and transcribed for further qualitative analyses. This research received approval from the Taipei Medical University Joint Institutional Review Board (approval no. N201603093), and all subjects signed an informed consent form.

### 2.4. Interview guide development

The semi-structured interview guide was developed from Leventhal’s CSM to reveal both PD patients’ and caregivers’ perspectives regarding the disease progression and medication therapy. The interview guide was divided into two parts; part A was the interview guide for the PD patients and part B for their caregivers. Part A included: (1) disease awareness, (2) medical care, (3) medication use of the PD patients. Disease awareness questions probed on disease confirmation (Q1), symptoms (Q2), mental outcomes (Q3), and others’ understanding of the disease (Q4). Medical care questions probed on patient’s awareness of their care (Q5), and patient’s anticipation of the medical environment (Q6). Medication use was probed on communication between doctors and patient (Q7), medication awareness (Q8), and their knowledge of medications (Q9). Questions in Part B aimed to understand the caregiver’s feelings (Q1), caregivers’ communication with patients (Q2), caregiver’s caring feelings (Q3), and caregivers’ anticipation of the medical environment (Q4). The above interview questions had been reviewed and revised by a neurologist, two clinical pharmacists, and a PhD researcher before the main data collection to ensure the quality of the contents.

### 2.5. Analysis

A qualitative content analysis was conducted using a directed approach (Hsieh & Shannon, 2005). Initial codes were established based on the CSM; as such, the interview guide partly informed the initial coding process. Then, one coder read and reread the interview transcripts, naming condensed meaning units as codes, grouping clusters of related codes into potential sub-themes, and further integrating them into themes. After the initial round of coding, the second coder reviewed the interview transcripts, themes and ensured that the messages had been conveyed without bias, continually comparing and merging the themes throughout the study progress until agreement was achieved. For ethical considerations, individual identities were transformed to codes in the transcripts. Researchers used the ATLAS.ti 7.5.6 (Berlin, Germany) qualitative software for the analysis. Patient and caregiver characteristics were analysed by descriptive statistics.

### 2.6. Trustworthiness

The trustworthiness of this study was established based on four criteria: credibility, transferability, dependability, and confirmability through the following strategies (Lincoln & Guba, 1985). To ensure the credibility of the study, the researchers recorded the participants’ responses, and read and reread the verbatim transcripts to vividly describe their experiences with associated quotations. To achieve transferability and dependability, we imported the interview transcripts into the above-mentioned qualitative software to dissemble the contents and vividly convey the responses of the participants through coding classification with associated quotations. Audit trails were maintained, including the original recordings and questionnaires as well as verbatim transcripts. Finally, study recruitment and interviews were conducted by the same investigator, who was a pharmacy student with knowledge of PD medications and who had undergone special training in qualitative methods and in-depth interview skills from a PhD‐trained researcher with expertise in qualitative studies. Regular discussions of the details of the study process were held to ensure the confirmability of the findings.

## 3. Results

In total, 15 interviews were conducted with a total of 15 patients with PD and eight caregivers. Characteristics of patients and their relationship with usual caregivers are shown in Table I. The majority of patients were male (73%); over half of the patients were 70 years or older, and had a high school education or less. All patients were receiving L-dopa medication, with the majority having begun this medication within the past 2 years. Five core themes were identified from PD patients’ and their caregivers’ experiences in this study (Table II).

**Table I.** Patient characteristics (N = 15)

Item	*n*(%)
Gender Male Female	11 (73.3) 4 (26.7)
Age (years) 50 ~ 59 60 ~ 69 70 ~ 79 80 ~ 89	4 (26.7) 3 (20) 6 (40) 2 (13.3)
Education Elementary school (and below) Junior high Senior high University Master’s degree or above	5 (33.3) 2 (13.3) 3 (20) 4 (26.7) 1 (6.7)
Current occupation Retired or none Service Industry	8 (53.3) 6 (40) 1 (6.7)
Disease duration (years) ≤2 3 ~ 5 6 ~ 10 16 ~ 19	9 (60) 3 (20) 2 (13.3) 1 (6.7)
Medications L-dopa Dopamine agonist COMT inhibitor MAO-B inhibitor Amantadine Anti-cholinergic	15 (100) 8 (53.3) 0 (0) 0 (0) 0 (0) 2 (13.3)
Duration of taking L-dopa <6 months 6–11 months 1–2 years 5 years 6 ~ 9 years 10 years	N = 14 1 (7.1) 1 (7.1) 7 (50.0) 3 (21.4) 1 (7.1) 1 (7.1)
Identity of usual caregiver Spouse Direct relative Spouse and direct relative Foreign worker	N = 14 10 (71.5) 2 (14.2) 1 (7.1) 1 (7.1)
Living with the usual caregiver	N = 14 14 (100)
Duration with usual caregiver (years) 2 5 6 ~ 9 16 ~ 19	N = 14 8 (57.1) 3 (21.4) 2 (14.2) 1 (7.1)
Support by usual caregiver Participates in treatment discussion Reminds to take medication only None of the above	N = 14 9 (64.3) 1 (7.1) 4 (28.6)

**Abbreviations**: COMT, catechol-o-methyl transferase; MAO-B: monoamine oxidase-B

**Table II.** Core themes and subthemes

Core themes	Sub-themes
1. Symptoms and help-seeking behaviours before a diagnosis	Experiencing PD symptoms Self-treatment before diagnosis
2. Emotional impacts and life adaptations of patients after a PD diagnosis	Feelings after the diagnosis Difficulties in public Changes and worries about work Changes in daily activities
3. Life affected by medications	Medication effects and perceived feelings Life readjustment and medication adherence in maximizing treatment effects
4. Experience of caregivers in taking care of PD patients	Becoming depressed Giving motion orders
5. Communication between doctors and patients	Reconciling doctor’s words with actual experience Social care in PD support groups

### 3.1. Theme 1: symptoms and care-seeking behaviours before a diagnosis

#### 3.1.1. Sub-theme 1-1: experiencing PD symptoms

PD patients experienced symptoms like dizziness, tremors, bradykinesia, stiffness, impaired balance, and memory loss, which were sometimes attributed by participants to stress or ageing and not easily recognized as PD symptoms before a diagnosis. Such confusion led to delays in seeking medical help.

Lots of primary symptoms …, the first was having nightmares, kicking and beating someone … shouting, and also dizziness. It was … not like just turning around, like when you get up from the bed, it was like doing a somersault. It was like in your head … your whole body was rolling … rolling forward like a somersault. Very strange.—Patient interviewee C

I can’t control my hands. The most obvious moment is when I brush my teeth. I don’t have enough strength when I need to brush in the horizontal direction. I am able to carry a bucket of water, but I can’t brush my teeth in the horizontal direction. Another moment is when I cook, I can’t raise my hand. It is [supposed to be] an ordinary movement, but now I just don’t have enough strength.—Patient interviewee D

#### 3.1.2. Sub-theme 1-2: self-treatment before diagnosis

Before going to a general practitioner, some of them had tried alternative therapies or physical therapy to improve the flexibility of the limbs; however, most of what the subjects tried provided limited improvement.

Alternative therapy … yes, I tried many times … However, it was not helpful. I tried folk therapy. I’ve tried other alternative therapies before being diagnosed with Parkinson’s disease, and they were all ineffective.—Patient interviewee I

I tried electric therapy; it makes me drooling … and finally I did not go there anymore.—Patient interviewee B

(For loss of limb muscle strength) I had tried physical therapy for almost a year, but symptoms persisted without much improvement—Patient interviewee D

### 3.2. Theme 2: emotional impacts and life adaptations of patients after a PD diagnosis

Living with PD as a chronic disease every single day, patients revealed the mental status from initially being diagnosed to facing changes and challenges in their daily lives, including in public, their work lives and daily activities. Through patients’ experiences, their knowledge of PD, feelings of living with the disease, and how they readapted to the society and to their daily lives were understood. Others’ perspectives and understanding of PD also impacted on patients’ attitudes towards their disease and lifestyle.

#### 3.2.1. Sub-theme 2-1: feelings after a diagnosis

After being diagnosed with PD, patients felt their future plans were ruined and wondered about the causes. Also, the inconvenience influenced patients’ willingness to go out in public. Some patients became extremely emotional and depressed.

Before retirement … I wanted to … I wanted to do something … and it’s all ruined.—Patient interviewee L

I feel extreme suffering from this disease. A lot of people with this disease have more-serious symptoms and suffer much more than me. It is very easy to come up with the idea of committing suicide.—Patient interviewee E

There are many potential causes of PD. However, it was hard for every individual patient to define actual causes. Therefore, patients searched through lots of information to get a better understanding of the disease, even though those ideas might not be based on current science. One patient suspected that his working environment at a young age might be the cause.

I assume that it is because of the working environment. I worked in a garage full of toluene. In addition, the stress, tension, and anger … resulted in my bad mood when I was treated badly by customers. They were taxi drivers.—Patient interviewee C

#### 3.2.2. Sub-theme 2-2: difficulties in public

Most patients had experienced inconveniences caused by the disease and had the sympathy of others in public. These situations affected their willingness to go outdoors. Caregivers also suffered mood swings because they worried about the occurrence of accidents when they were not around. Caregivers indicated that uneven roads and narrow sidewalks were dangerous for disabled or elderly patients. Furthermore, some patients would rather not go travelling anymore since “freezing” in public put patients in danger and caused extreme anxiety.

I do not feel like attending an event. The feeling, the feeling is like … why have I become so sick like this … Right now as I speak, I feel it is so different from before … I was very active, thought and talked very fast. Now every part of me is slow.—Patient interviewee C

Sometimes it’s inconvenient to go upstairs to a restaurant. Because my hands are shaking, it’s not practical to use chopsticks. I use spoons instead at home. But not every restaurant is equipped with spoons. I worry about this if I need to meet my friends in a restaurant. I am not comfortable when many people are around … I feel odd. I am like a sick man sitting there. So, I rarely hang out now.—Patient interviewee A

He is anxious. When going into a lift or getting on a bus, he sometimes stops right there with his body trembling … When I got off the metro, he suddenly froze inside the train. I went back to take him out. Otherwise it was difficult for him to get out like that.—Caregiver interviewee E

I am afraid of being on a business trip or traveling alone. The fear is … for instance, when I arrive at my destination, I can’t get off the transportation vehicle arbitrarily when I lose control over my body.—Patient interviewee M

#### 3.2.3. Sub-theme 2-3: changes and worries about work

Some patients believed that their symptoms affected their ability to work. Losing one’s job and the impending financial crisis were issues of great concern to patients. Changes in their work performance are an inevitable consequence of PD symptoms, and that hindered patients from revealing their condition in the workplace. Patients were bothered by lower efficiency and interrupted thinking processes after they suffered from PD, which contributed to their stress and anxiety about their job.

I need colleagues to back me up. I can’t do it by myself (sigh) … Everyone gossips. That’s why I try not to talk about my disease. I have been working in the industry for more than twenty years. I am afraid that I will be fired.—Patient interviewee I

Though I can read every word, it takes me a long time to underhand the whole sentence. The time and the degree of understanding is much worse than before …, so I might not be able to work. I know this situation will be worse.—Patient interviewee D

I realized that I can’t instantly understand the meaning of words. It is a feeling of disconnection and my thoughts gets stuck. I need to think over and over again. … However, I need to handle several steps at the same time when working. It’s necessary to instantly come up with subsequent steps when I decide what the first step is.—Patient interviewee D

#### 3.2.4. Sub-theme 2-4: changes in daily activities

Patients may encounter problems at home which changing their life patterns. To overcome these challenges, both patients and caregivers made efforts to improve patient self-management. Caregivers played a critical role in reminding patients about changing their pace, and they kept an eye on their patient. To make some adjustments to their daily lives, patients came up with some self-made devices to help themselves complete daily activities without others’ assistance. Some patients realized the importance of exercising after they were diagnosed with PD. Knowing the pain of stiffness without exercise, patients stated that exercising along with medication therapy was helpful in relieving the symptoms.

Sometimes he can’t even move or walk. When he hurries to go to the restroom, he needs to crawl or he can’t get there in time. To move faster, we have a wheelchair ready at home, but he doesn’t like it … One or two times he didn’t make it to the restroom in time.—Caregiver interviewee E

So anxious! Wanna pee, but I just can’t walk. So, I crawled and peed in my pants.—Patient interviewee E

There is a table in my bedroom with four legs. I tied a rope from my bed to one of the legs, and then tied a lot of knots so … I can try to sit up by myself. I used to sleep on my side and it was not convenient when I got up … Now it’s easier to get up from the side.—Patient interviewee M

I began practicing qigong after being diagnosed with PD … It’s necessary to do sports; otherwise, I get sore and can’t move. My body becomes very stiff after lying down. Now, my joints get more flexible after exercising. It’s very painful to move if I am stiff.—Patient interviewee E

### 3.3. Theme 3: life affected by medications

After seeking medical advice, patients faced the issue of readjusting to living with the disease and taking medications. Taking medications played significant roles in PD patients’ experiences with various stages of the disease progression. Patients’ feelings, medication effects, and impacts on their lives at various PD stages were investigated.

#### 3.3.1. Sub-theme 3-1: medication effects and perceived feelings

Medication effects contributed to patients’ relief from physical symptoms and also improvements in QoL. Patients reported a wider range of movement as perceived effectiveness of their medications. Owing to relief of symptoms, medications were regarded as patients’ independence and relief from other’s assistance when engaged in daily activities.

No tremors. Like writing, my fingers become nimble. My handwriting used to be big, then it got smaller with progression of the disease. After taking XX [medication L-dopa], my fingers can stretch out wider. The movement range is bigger and not as rigid as before … not exactly getting back to normal but still a big improvement. The distance of each footstep is not so small anymore.—Patient interviewee D

Less dizziness, so I feel better and I am able to go grocery shopping, cook, and go outside. It was very inconvenient when I felt dizzy outside by myself. But now, it’s alright even if I go out alone.—Patient interviewee O

#### 3.3.2. Sub-theme 3-2: life readjustment and medication adherence in maximizing the treatment effect

The side effects of medications impacted many aspects of patients’ lives during long-term therapy, especially concerning work and daily activities. Some patients rescheduled their dining time to maximize the medication’s effects according to their experience. However, shortened response to medication which necessitated readjusting their daily schedule greatly concerned patients. In order to achieve the desired effectiveness of medications, patients’ understanding of chronically and regularly taking medications reflected their medication adherence.

If I want to go for a walk in the park, it’s necessary to know if the medication’s effect will wear off when I am still there. In that case, I need to take the medications in the library close to the park and wait for its onset.—Patient interviewee M

To me, taking medications on time is nothing. It is like taking a course at school.—Patient interviewee F

### 3.4. Theme 4: experience of caregivers in taking care of PD patients

#### 3.4.1. Sub-theme 4-1: becoming depressed

Due to the impaired mobility attributable to PD, caregivers are the people patients can rely on. However, during the caring experiences, caregivers also need to adjust their mood not only to confront the challenges of daily care routine, but also to satisfy patients’ mental needs.

The worse his disease becomes, the worse my mood is. Maybe one day I will have depression … I feel frustrated when he “freezes”. I find it very ridiculous when I just want to check if he’s alright and I will be blamed for it. He had a hard time because of drooling and slurring, so he didn’t want me to talk to him.—Caregiver interviewee E

#### 3.4.2. Sub-theme 4-2: giving motion orders

Caregivers also indicated that patients could take big steps if tips on addressing difficulties in their daily lives were found, which can help patients walk normally again as soon as possible.

I need to give him orders. Ask him to raise his leg, and then he will sort of begin to move. The second step is easier after the first step is achieved. It seems like a power button that the nerves start to work when one of the leg lifts up.—Caregiver interviewee E

### 3.5. Theme 5: communication between doctors and patients

#### 3.5.1. Sub-theme 5-1: reconciling doctor’s words with actual experience

Patients’ medication-taking behaviours were related to the communication and relationship between the patient and their physician. According to interviewees’ descriptions, patients’ perspectives of the physician-patient relationship and interactions with patient-support groups were explored. Patients tended to believe information regarding the disease and medications provided by their physician, and revealed their concerns and worries about disease progression, which showed a solid physician-patient relationship. Still, patients mentioned their long-term expectation for PD treatment was to recover from the disease and counted on researchers’ further studies to look for a solution to PD.

I’ve talked to the doctor. He said that PD will impair my conceptual response. I asked him if it was like Alzheimer’s disease and he said it was not the same. The doctor asked me not to worry. I was anxious because I needed to deal with a lot of things at work …—Patient interviewee D.

#### 3.5.2. Sub-theme 5-2: social care in PD support groups

Patients reported positive attitudes towards patient-support groups which included patients and physicians. Via social media groups fostering conversations and connections among members, patients were able to ask questions or share their own experiences with others in need. In addition, physicians could answer patients’ questions in a timely manner and convey correct information about self-management of PD.

PD support groups help. The group of patients and doctors on LINE really helps me a lot. In this group, we can share good ideas and experiences, and also ask questions. Furthermore, when anyone needs help, people in the group can offer assistance and provide relevant information.—Patient interviewee M.

## 4. Discussion

This study investigated subjective experiences of living with PD and emphasized daily adaptations after taking medications from both the patients’ and caregivers’ perspectives. Faced with impacts on daily activities, work life, and social life caused by PD, patient attitudes towards the disease and the meaning of medication use changed over the time. The coping and accommodative strategies of living with PD may differ from culture to culture, as in Chinese populations, the naturalistic philosophies of Buddhism, Confucianism, and Taoism may result in patients’ acceptance and ability to make peace with their PD-related disabilities rather than fighting against the disease (Kwok et al., 2020).

Although care burdens led to depression among caregivers, they developed coping strategies to address difficulties while caring for PD patients. Furthermore, timely and reliable provision of disease and medication information facilitated strong relationships and interactions between patients and healthcare professionals.

Various medication experiences influencing patients’ medication-taking behaviours were found in this study, including the length of medication use, the medication-taking schedule, side effects, and psychosocial factors. These findings coincided with previous observations, which suggested that patients in the late stage of PD need to follow a fixed daily schedule to avoid the inconvenience resulting from the medication wearing-off (Groenendaal et al., 2010; Hasson et al., 2010). The vivid patient experiences of difficulties encountered captured in this study can help the society better understand PD patient needs.

Symptom burdens and unmet needs in PD patients are described in the literature, which might also be influenced by cultural factors; Kwok et al. described the unmet needs of Asian PD patients, and mentioned the physical needs and emotional aspects are the most problematic for Thai patients, whereas patients from Hong Kong and Taiwan emphasized psychosocial and spiritual support (Kwok et al., 2021). In our study, many patients shared their negative feelings at being diagnosed with PD. Losing strength for a patient is like losing the relationship between their mind and body. Some patients without a family history could not figure out the cause of the disease. Some patients were worried about the consequences of PD, including financial burdens, disrupted future plans, and social embarrassment in public, further leading to personality changes, social anxiety, and even depression. Clinically speaking, depression in patients with PD could be related to dopaminergic neuron degradation (Kritzinger et al., 2015). However, patients in our study also embodied the value of regular exercise, which motivated patients to step outside and led to symptom improvement. This coincides with previous studies which showed that regular exercise and mind-body interventions such as mindful yoga, aerobic exercise, dancing, boxing, and tai chi might improve PD patients’ motor symptoms, and also psychological aspects such as depression, anxiety, isolation from society and their life quality (Chen et al., 2020; Kampling et al., 2019; JY Kwok et al., 2016; Kwok et al., 2019; MacCosham et al., 2019). A study also summarized two reasons that inspired patients to exercise: having a specific purpose for their lives and becoming confident in their own ability (Eriksson et al., 2013). These findings can remind healthcare professionals and caregivers to keep patients’ mental status in mind when evaluating and caring for patients. To ensure a better QoL, exercising regularly may help patients regain confidence in life; interventions from social workers and counsellors may be introduced if needed.

With regard to interactions among PD patients, patients felt connected when they were free to share and ask questions in patient groups. A previous study also reported that self-help groups helped them accept the disease and become better satisfied with their daily lives (Charlton & Barrow, 2002). However, other research found that patients might be frightened when seeing other patients who are going through the latter stages of PD, as they regarded others’ situations as their own future consequences. Furthermore, being a part of an underprivileged group caused patients to lose their dignity (Tan et al., 2012). Therefore, to encourage patients’ positive interactions in support groups, the involvement of healthcare professionals is necessary to lead appropriate information exchanges and facilitate medication adherence and healthy lifestyles.

Caregivers in this study mostly reported on patients’ daily lives, patients’ psychological status, and support provided by the caregivers; however, a few of them mentioned their emotions about caring for PD patients, showing their depressive feelings and helplessness due to patients’ negative emotions as the disease progressed. The literature shows that cognitive impairment is not only strongly associated with a lower QoL of patients and caregivers, but also predicts caregivers’ burdens (Corallo et al., 2017). The majority of caregivers in this study were patients’ spouses who were also at an older age. This situation reflects the potential issues of long-term senior care, such as spouses’ absence or cognitive impairment (Armstrong et al., 2019). Previous research also investigated issues of concern to caregivers, including irremovable responsibility, inadequate information about caring, and the needs of support from the healthcare system and society (Tan et al., 2012). Our research showed that some caregivers gained insights into the disease online and understood even more than the patients themselves. However, inconsistent and insufficient information from physicians resulted in anxiety and insecurity (Giles & Miyasaki, 2009). A prospective study interviewing PD caregivers emphasized the importance of engaging and educating caregivers to promote patient-centred care (Rastgardani et al., 2019). Therefore, sufficient information about the disease provided by healthcare professionals, understanding and assistance from others in public places, and psychological support for caregivers might reduce their burden and improve the QoL of both patients and caregivers.

Adequate communication between patients and physicians led to reinforcement of medication information, psychological support, effective problem-solving, and satisfaction. Patients in this study took comfort from being counselled by physicians, due to the physicians’ detailed explanation of the disease and medications, as well as dosage adjustments that addressed the patients’ fear of dementia and physical discomfort. Some patients even completely followed their physicians’ instructions based on absolute trust. This finding is contrary to a previous study which indicated that a lack of education and information regarding PD provided by healthcare professionals and insufficient emotional support led to patients’ dissatisfaction (Boersma et al., 2016). Patients in this study were also faced with difficulties communicating with physicians; however, many developed their own solutions. For instance, to avoid missing questions they wanted to ask, patients listed their problems in advance due to limited time during the clinic visit. This finding is supported by prior research which identified communication barriers (Armstrong et al., 2019; Schrag et al., 2018). Therefore, understanding patients’ needs and providing appropriate timely solutions or adequate educational materials might improve communication between patients and physicians, and further lead to better treatment satisfaction.

There are limitations of this research. First, enrolment of participants from a single centre may limit the generalizability of the study. Second, since the majority of our participating patients were taking L-dopa (100%) and dopamine agonists (53.3%), the experiences they provided were mainly caused by these two classes of PD drugs and might not be generalized to patients who take other classes of PD medications. Third, the influence of patients’ cognitive impairment stage, depression evaluation, and comorbidities were not investigated in this study. The quotations should be interpreted only as subjective experiences rather than a full clinical picture. Finally, some patients and their caregivers were interviewed together, which might have limited a full revealing of their deeper feelings. However, vivid descriptions from individual participants were retained and provided rich experiences.

## 5. Conclusions

This study explored patients’ and caregivers’ experiences of living with PD and coping with medication use. PD disease progression and medication use caused patients to adjust their daily schedules and personal activities. Life adaptations regarding work life, social life, and daily activities due to PD disease progression and medication-related problems affected patients’ and caregivers’ QoL. Patient support groups involving caregivers and healthcare professionals are recommended to strengthen connections for better care of patients with PD. Interventions providing sufficient patient education, timely mental support, and adequate communication with healthcare professionals might efficiently address concerns derived from patients’ and caregivers’ experiences and further improve patients’ QoL.

## Appendix. Semi-structured interview guide

**A. Interview guide for PD patients**

**Table:**

**Disease awareness** How did you realize you had the disease? What is the difference in your body compared to the old days? Since you discovered something was wrong with you, what is the difference in your daily life? Have you made any adjustments to your daily life? What kind of changes have you had so far since you were diagnosed with PD? Do you encounter difficulties in public areas? What was that experience? Could you share with us? What did it mean to you when you were diagnosed with Parkinson’s disease? What was the impact on your daily life? What did others think of you?
**Medical care** On what occasion did you seek for others’ help? What was your feeling? What would you like to know about the disease? What do you hope will improve by the medical environment and policy?
**Medication use** Would you please share your experience discussing your disease with doctors in the clinic? What is the difference between before and after taking your medications? Could you share the impacts of the medications on your daily life and personal feelings? Did you have the experience of asking your doctor to change the prescription? Could you share the experience when a doctor changed your medications in the past?

**B. Interview guide for caregivers**

**Table:**

**Medical care** When do you give assistance to patients? Under what situation? Using what method? Did you guide the patient or let him/her help himself/herself? Do you discuss caregiving with your patient? When do you feel frustrated? When do you have a sense of achievement? Could you share the experiences with us? What would you like to know about the disease? What do you hope will improve in terms of the medical environment and policy?
